# Effects of quality of dictionary in knowledge-based 6-plane automatic slice-alignment method for cardiac magnetic resonance imaging

**DOI:** 10.1186/1532-429X-14-S1-P268

**Published:** 2012-02-01

**Authors:** Shigehide Kuhara, Shuhei Nitta, Tomoyuki Takeguchi, Nobuyuki Matsumoto, Kenichi Yokoyama, Masamichi Imai, Rieko Ishimura, Toshiaki Nitatori

**Affiliations:** 1 MRI Systems Development Department, Toshiba Medical Systems Corporation, Otawara-shi, Tochigi, Japan; 2 Corporate Research & Development Center, Toshiba Corporation, Kanagawa, Japan; 3 Department of Radiology, Kyorin University, Faculty of Medicine, Tokyo, Japan

## Summary

We have developed a new automatic slice-alignment method that employs a combination of knowledge-based algorithm and model based algorithm to determine six planes at the same time. We have also evaluated the effects of the quality of the dictionary used in this method.

The results showed that the robustness of slice determination in the proposed method can be improved as compared to the model-based only algorithm by using an appropriate dictionary and can be further improved if a larger number of patient datasets is used.

## Background

We have developed a new automatic slice-alignment method that employs a combination of knowledge-based algorithm and model based algorithm to determine six planes at the same time. We have also evaluated the effects of the quality of the dictionary used in this method.

## Methods

ECG-gated 2D steady-state free precession (SSFP) axial multi-slice images covering the entire heart area were acquired using a 1.5-T MRI scanner (Excelart Vantage ^TM^ Powered by Atlas, Toshiba Medical Systems) during a single breath-hold. A total of 17 healthy volunteers (37 datasets) and 35 patients (36 datasets) were acquired. After several features of the heart (e.g., the mitral valve and cardiac apex) were identified using a knowledge-based algorithm, a total of six planes were determined (vertical long-axis, horizontal long-axis, short-axis, 4-chamber, 2-chamber, and 3-chamber views) by employing the slice alignment procedures according to SCMR protocols. The quality of the dictionary was changed as follows: 1) no dictionary (model-based algorithm), 2) dictionary of volunteers only (12 datasets), and 3) dictionary of both volunteers (12 datasets) and patients (27 datasets). The number of datasets used for the dictionaries was also changed from 0 to 13 (5 volunteers +8 patients), 26 (9 volunteers +17 patients), and 39 (12 volunteers +27 patients). Robustness and accuracy were evaluated by calculating the success rate for plane detection and the angular error from the 'answer' position determined by two physicians. The evaluation was performed as the evaluated dataset itself was not included in the dictionary.

## Results

**Figure 1:**
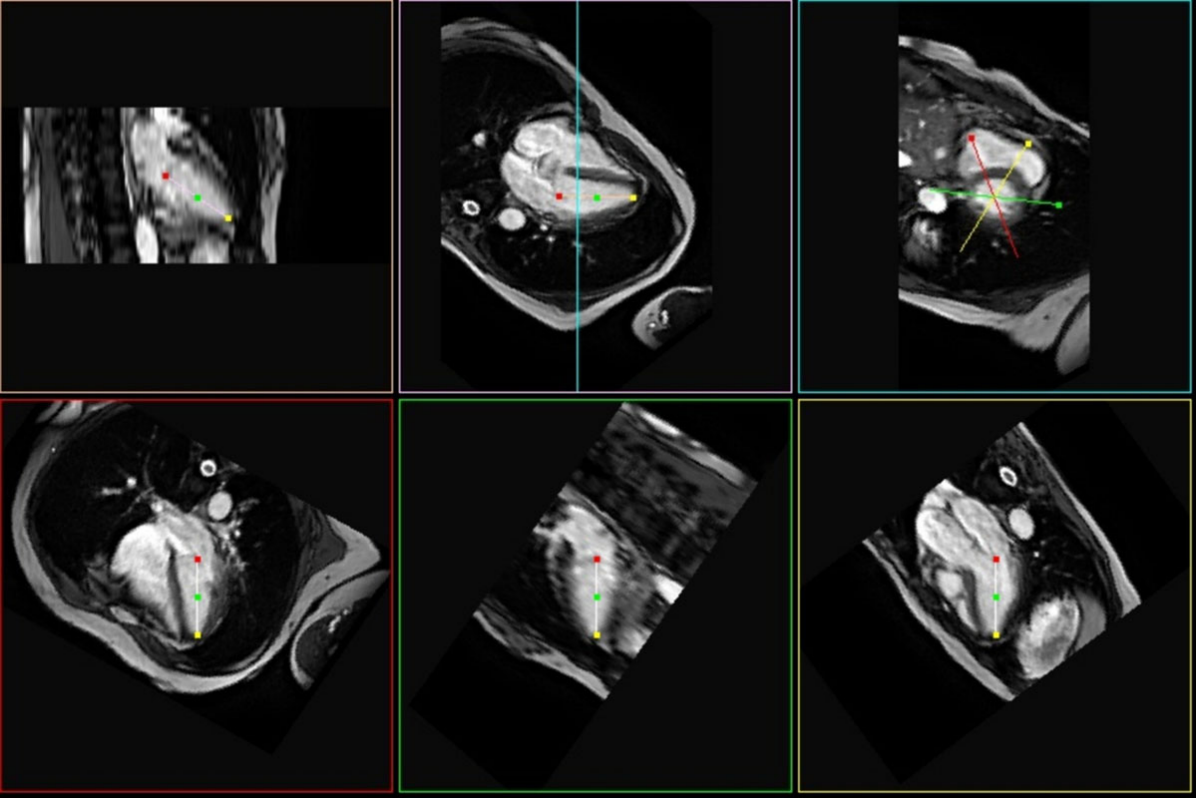
Example of knowledge-based 6-plane automatic slice alignment in a patient.

The proposed method worked well even in the patients, permitting all six planes to be determined at the same time (Figure 1). The success rates were 82%, 97%, and 100% for the total of 73 datasets and the mean angular error was reduced from 10.2±12.1° to 5.3±4.8°, and 4.1±2.7° in cases 1), 2), and 3), respectively, indicating that the use of a more practical dictionary improved the success rate and reduced the mean angular error. The success rate was also increased from 82% to 97%, 99%, and 100% and the mean angular error was reduced from 10.2±12.1° to 5.0±5.1°, 4.4±4.1°, and 4.1±2.7° as the number of datasets used for the dictionary was increased from 0 to 13, 26, and 39.

## Conclusions

The robustness of slice determination in the proposed method can be improved as compared to the model-based only algorithm by using an appropriate dictionary and can be further improved if a larger number of patient datasets is used.

## Funding

No funding was received for this research.

